# Concepts of Regeneration for Spinal Diseases in 2021

**DOI:** 10.3390/ijms22168356

**Published:** 2021-08-04

**Authors:** Takashi Yurube, Inbo Han, Daisuke Sakai

**Affiliations:** 1 Department of Orthopaedic Surgery, Graduate School of Medicine, Kobe University, 7-5-1 Kusunoki-cho, Chuo-ku, Kobe 650-0017, Japan; 2 Department of Neurosurgery, School of Medicine, CHA University, CHA Bundang Medical Center, Seongnam-si 13496, Gyeonggi-do, Korea; hanib@cha.ac.kr; 3 Department of Orthopedic Surgery, School of Medicine, Tokai University, 143 Shimokasuya, Isehara, Kanagawa 259-1193, Japan; daisakai@is.icc.u-tokai.ac.jp

It is our pleasure to announce the publication of the Special Issue “Regeneration for Spinal Diseases” in the *International Journal of Molecular Sciences* (IJMS, ISSN 1422-0067). Spinal diseases place a significant burden on the general population. The prevalence of degenerative spinal diseases, including intervertebral disc degeneration and osteoporosis, is quite high and increases with age, and the affected population may suffer from a long-term disability. Furthermore, the most serious spine-related conditions can reach spinal cord injury due to traumatic or non-traumatic causes. Despite recent advances in the management of these common but problematic spinal pathologies, there is growing research interest in discovering novel therapeutic strategies.

This IJMS Special Issue “Regeneration for Spinal Diseases” includes 10 cutting-edge original research articles consisting of six papers for degenerative disc disease and four for spinal cord injury. Moreover, five expertized review articles targeting the intervertebral disc, neuropathic pain, pyogenic spondylodiscitis, and surgical bone fusion have been compiled. All of these 15 articles should provide helpful insights for the current clinical management and future basic science development of regenerative treatment strategies for intractable spinal diseases.

These articles have been published in the IJMS (https://www.mdpi.com/journal/ijms, 1 August 2021), Section: Molecular Pathology, Diagnostics, and Therapeutics (https://www.mdpi.com/journal/ijms/sections/Pathology_Diagnostics_Therapeutics, 1 August 2021), Topic: Regeneration for Spinal Diseases (https://www.mdpi.com/journal/ijms/special_issues/Spinal_Diseases, 1 August 2021). The IJMS is an international, peer-reviewed, open access journal published by the Multidisciplinary Digital Publishing Institute (MDPI), universally and freely accessible online.

## 1. Degenerative Disc Disease

With the global trend of aging, low back pain is a worldwide health problem because of its enormous morbidity and socioeconomic strain. The etiology of low back pain is largely non-specific; however, lumbar disc degeneration is the main risk factor for disabling low back pain. Intervertebral disc degeneration can develop not only back pain but also neurological disorders including radiculopathy, myelopathy, paralysis, and bladder and bowel dysfunction. Currently, surgical resection and/or fusion are the primary treatment for degenerative disc disease, resulting in the loss of function including shock absorption and spinal movement. Therefore, the development of regenerative therapies for intervertebral disc degeneration is a high requirement.

The intervertebral disc has a complex structure consisting of the central nucleus pulposus (NP) and the peripheral annulus fibrosus (AF) and cartilage endplates. The disc AF and NP originate from the mesenchyme and notochord, respectively. Adolescent notochordal cell disappearance in the disc NP is speculated to be an early hallmark of degeneration, as morphological and biochemical disc degeneration starts from early childhood. This would also have a possible link with an extremely low nutrient, severe hypoxic environment of the disc, the largest avascular organ in the human body.

Kim JW et al. [1] conducted a comprehensive literature review regarding the involvement and roles of hypoxia-inducible factor-1alpha (HIF-1α) in intervertebral disc degeneration. The HIF-1α is a master transcription factor that initiates a coordinated cellular cascade in response to low oxygen tension, which is then required for disc NP development and homeostasis. Recapitulating prior reports, this review suggests HIF-1α as an early diagnostic marker and therapeutic target against degenerative disc disease.

Recent works by a Guest Editor, Takashi Yurube’s laboratory have focused on autophagy in the intervertebral disc. The below studies both suggest the modulation of autophagy as a potent molecular treatment strategy for degenerative disc disease. Ito M et al. [2] tested inhibitory effects of autophagy at the early stage by RNA interference of autophagy-related gene 5 (ATG5) and at the late stage by pharmacological chloroquine supplementation in human degenerative disc cells surgically obtained. This in vitro loss-of-function study at different stages by ATG5 knockdown and lysosomotropic chloroquine clarifies consistent human disc cellular protection of autophagy against apoptosis and senescence rather than against extracellular matrix catabolism.

Yurube T et al. [3] observed in vivo autophagy impairment in NP notochordal cells and apoptosis induction in NP non-notochordal cells under unphysiological mechanical loading-induced experimental disc degeneration of rat tails, supporting the interpretation that autophagy could protect notochordal cell homeostasis through limiting apoptosis in the disc NP.

Bovine intervertebral discs are an important cell source to study the pathophysiology because of the similarity in the phenotype to humans. Calio M et al. [4] performed data analysis of single-cell RNA sequencing and bulk RNA sequencing in young, healthy bovine tail discs, identifying 27 NP structure/tissue-specific genes and 24 AF structure/tissue-specific genes. In this study, notochordal-like and progenitor stem cell-like NP-cell clusters and stem cell-like, fibroblast-like, and endothelial progenitor cell-like AF-cell clusters were detected.

Takeoka Y et al. [5] studied bovine tail disc NP cells incubated with chondroitin sulfate proteoglycan or hyaluronan under hydrostatic pressure mimicking spinal motion. Chondroitin sulfate proteoglycan had anabolic effects on cells combined with hydrostatic pressure, which could be a potential therapeutic and regenerative scaffold for degenerative disc disease.

Progenitor and/or stem cell transplantation has been a long-debated treatment strategy to regenerate intervertebral disc tissues. Croft AS et al. [6] conducted a narrative literature review about the effectiveness of mesenchymal stromal cell (MSC)-based therapies for degenerative disc disease. Preclinical in vitro, ex vivo, and in vivo studies presented the development of MSCs with a disc cell-like phenotype and of hydrogels and scaffolds with embedded MSCs. Clinical studies further reported the improvement of low back pain symptoms and magnetic resonance imaging degenerative appearances following intradiscal MSC injection. In addition, the most promising, alternative approach to intradiscal cell injection seems to take advantage of MSC homing capabilities.

Friedmann A et al. [7] performed a 1-year follow-up study to assess the regenerative potential of percutaneous injection of an autologous adipose-derived stem cell-loaded hydrogel comprising bovine collagen types I and II in a sheep model of nucleotomy-induced disc disruption. This preclinical study did not unfortunately reach marked quantitative differences of tissue repair in computed tomography and histology, which however provides an effective translational approach for degenerative disc disease.

A Guest Editor, Daisuke Sakai’s laboratory identified angiopoietin-1 receptor (Tie2)-positive human intervertebral disc NP progenitor cells with multipotency and high self-renewal abilities, which can be available for a potent cell source for regenerative cell therapies against disc degeneration. However, it is difficult to enhance and maintain these cells in standard monolayer culture systems. Sako K et al. [8] therefore tested the efficacy of whole tissue cultures combined with fibroblast growth factor 2 supplementation using human young herniated disc NP tissues, which increased Tie2 maintenance through the extracellular signal-regulated kinase (ERK)/mitogen-activated protein kinase/ERK kinase and to a lesser extent phosphatidylinositol-3 kinase/Akt pathways. These techniques are useful for human disc NP progenitor cell expansion and experimentation. In addition, Tie2 has been suggested as a marker of collagen type II-producing cells.

## 2. Spinal Cord Injury

Spinal cord injury is a devastating condition of the damage to the spinal cord primarily caused by trauma but also by vascular disease, infection, and tumors, which can induce temporary or permanent motor, sensory, and autonomic dysfunction and associated complications. Currently, effective treatments are very limited and largely symptom-relieving and further deterioration-preventing, e.g., corticosteroid administration (controversial), decompression and stabilization, and rehabilitation. Therefore, there is a great need to develop new therapeutic strategies to facilitate neurological recovery and tissue repair. In particular, stem cell therapy is one of the most promising treatment options for spinal cord injury.

Chang DJ et al. [9] assessed the efficacy of genetically modified human neural stem cells from telencephalon tissues in a 15-week gestational fetal brain overexpressing brain-derived neurotrophic factor (BDNF) (F3.BDNF) in a rat model of spinal cord injury. Intrathecally transplanted F3.BDNF cells migrated toward the injured spinal cord area, expressed neural lineage markers, and had connection to the host neurons. Then, F3.BDNF-transplanted rats exhibited improved locomotor function, histologically increased spared myelination and decreased cystic cavity, and reduced numbers of inflammatory cells and astrocytes. The presented findings suggest that the transplantation of F3.BDNF cells can modulate inflammation, glial activation, and hyperalgesia following spinal cord injury.

Lee HL et al. [10] reported the isolation of peripheral nerve-derived stem cells (PNSCs) from common iliac nerve segments harvested after brain death from adult human organ donors with similar characteristics to neural-crest stem cells, which was further enhanced by spheroid formation. In in vivo testing using a rat model of spinal cord injury, local transplantation of PNSC spheroids survived at least three weeks in part, induced functional recovery and neuronal regeneration, and reduced neuropathic pain, suggesting a new therapeutic approach for patients with spinal cord injury.

Won JS et al. [11] investigated in a rat spinal cord injury model the optimal injection route and dose for adult human multipotent neural cells (ahMNCs) from patients with hemorrhagic stroke. Within 24-h migration to the spinal cord lesion, over 5-week survival of lateral ventricle-transplanted ahMNCs were found. Then, promoted locomotor recovery was determined by the transplantation dose of ahMNCs with the minimum requirements. Histologically, ahMNCs exerted favorable effects through the modulation of glial scar formation, neuroprotection, and/or angiogenesis. Information on the successful indirect injection and optimal transplantation dose of stem cell therapy using ahMNCs could be clinically relevant.

Bighinati A et al. [12] evaluated post-traumatic expression profile of 100 extracellular matrix-related genes in the spinal cord segments rostral and caudal to the lesion of a rat spinal cord injury model. Consequently, injury-induced asymmetric expression with a higher regulation in the rostral segment of genes involved in matrix remodeling, adhesion, and cell migration was observed. Bioinformatics and protein analysis identified tissue inhibitor of metalloproteinases-1 and CD44, a hyaluronan receptor, as hub genes during post-injury, indicating matrix regulation outside of the lesion in spinal cord injury.

## 3. Neuropathic Pain

Neuropathic pain is a complex, heterogeneous, chronic disease condition affecting the somatosensory nervous system. The pathomechanism of neuropathic pain involves a wide range of molecular pathways. Specifically, neuroinflammation plays a critical role in the development and maintenance of neuropathic pain. However, current pharmacological and nonpharmacological, e.g., spinal cord stimulation, treatments of neuropathic pain are largely palliative and temporarily effective without substantial recovery.

Joshi HP et al. [13], a Guest Editor, Inbo Han’s research group, provided a large-scale literature review summarizing the efficacy of mesenchymal and neural stem cell therapies for neuropathic pain, based on a growing number of preclinical and clinical trial findings of arresting degenerative processes and promoting nerve survival and recovery. The fast onset and long-period effectiveness of stem cells are the principal advantage. The administration route of stem cells is also important. While local delivery is often used but at risk of adverse effects, e.g. tissue injury, systemic delivery is attractive because of the superior biodistribution, despite passive entrapment of circulating stem cells within tissues.

## 4. Pyogenic Spondylodiscitis

Pyogenic spondylodiscitis appears to develop advanced vertebral osteolysis and spinal destruction particularly in elderly and/or immunocompromised patients, which can cause severe back pain and disability as well as serious neurological problems including irreversible paralysis. While osteoporosis treatment has been well studied, therapies for infection-induced spinal bone loss still remain uncovered.

Ohnishi T et al. [14] experienced a patient case of osteolytic pyogenic spondylodiscitis successfully treated with romosozumab and sensitive antibiotics, which raised the need for an extensive literature review of molecular signaling pathways involved in infection and currently available osteoporosis treatment options against bone defect. Based on accumulating evidence, underlying pathophysiology of bone loss in older patients with pyogenic spondylodiscitis recommends the application of anabolic agents including romosozumab and teriparatide.

## 5. Surgical Bone Fusion

As a result of developmental and degenerative spinal disorders, lumbar spondylosis, spondylolisthesis, and spondylolysis can cause severe nociceptive low back pain and also intractable neuropathic complications. Lumbar interbody fusion is a surgical intervention to stabilize segments including disc and facet joint abnormalities as well as to directly and/or indirectly decompress neural components including the dural tube and nerve roots, ultimately providing favorable clinical and radiological outcomes.

Lo WC et al. [15] performed a narrative literature review regarding materials and technologies available to improve bone grafting during minimally invasive transforaminal lumbar interbody fusion surgery. Iliac crest bone autograft and allograft, demineralized bone matrix, ceramics including hydroxyapatite, cell-based regenerative therapies including stem cells, cellular bone matrices, blood-derived biomaterials and platelet growth factors, and platelet-rich plasma, synergistically assisted with three-dimensional printed cages, have been described to achieve better surgical outcomes.

## 6. Conclusions

In conclusion, we would like to declare the future regathering and recomposing of further successful, updated Special Issues, e.g., “Regeneration for Spinal Diseases 2.0”.
